# Seasonal Variations in Triptan Prescription in Japan: A Nationwide Time‐Series Analysis

**DOI:** 10.1002/brb3.70184

**Published:** 2024-12-22

**Authors:** Muneto Tatsumoto, Koichi Hirata, Takeo Nakayama, Kentaro Yamato, Hiromi Sano, Lyo Inuyama

**Affiliations:** ^1^ Headache Center Dokkyo Medical University Hospital Tochigi Japan; ^2^ Department of Neurology Dokkyo Medical University Tochigi Japan; ^3^ Department of Health Informatics School of Public Health Kyoto University Kyoto Japan; ^4^ Department of Medical Affairs Otsuka Pharmaceutical Co. Ltd Tokyo Japan

**Keywords:** migraine, observational study, seasonal, triptans, weather

## Abstract

**Objective:**

This study aimed to investigate seasonal differences in migraine onset in Japan and associated trends in the prescription of triptan.

**Background:**

The relationship between seasonal changes and the onset of migraine remains largely unknown.

**Methods:**

We combined the large‐scale medical claims data in Japan with city‐level meteorological data presented by the Japan Weather Association. The study period was from January 2018 to December 2019. We included patients aged 18–74 years and diagnosed with migraine who had been prescribed triptans in 2018. Patients were categorized into four groups according to the seasons when they were prescribed medicines: winter (January–March), spring (April–June), summer (July–September), and autumn (October–December). Migraine onsets were defined by the time of triptan prescription. The least‐square mean differences between the seasons were estimated with linear mixed‐effects models for repeated measures, adjusting for the following covariates: sex, age, acute migraine medications, and medications for migraine prevention, comorbidities that can induce migraine, other comorbidities, and the estimated age of menopause in females.

**Results:**

We analyzed data of 12,986 patients in 2019 after triptan had been prescribed (female 74.1%). The mean age was 44.1 years. The mean number of amounts of prescriptions comprised 2.12 tablets/month in spring and summer, whereas there were only 2.09 tablets/month in autumn and 2.00 tablets/month in winter. The amount of triptan prescribed in winter was lower than in spring (*β* = −0.117; 95% confidence interval [CI]: −0.169 to −0.065).

**Conclusion:**

The study results suggest that seasons can have an impact on the onset of migraine; thus, clinicians may advise patients to take preventive actions, including self‐care and drug therapies, from the winter stage. This should be done in order to reduce the number of migraine onsets in high‐risk season (spring) regardless of region.

## Graphical abstract

The results of this study suggest that seasons can impact the onset of migraine. Thus, to reduce the amount of triptan prescriptions, clinicians may advise patients to take preventive actions, including self‐care and drug therapies from winter to reduce the number of migraine onsets in spring regardless of region.

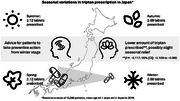

## 1 Introduction

Migraine is a common neurological disease that affects people around the world and is a major cause of disability among males and females (Agosti 2018; Lipton et al. 2007). In Japan, 6.0%–8.9% of the population have been reported to suffer from migraines, which has a significant negative effect on their quality of life (Igarashi et al. 2020, Takeshima et al. 2004). The prevalence of migraines in Japan is higher than that in other countries, and this high rate is attributed to weather changes and hormonal imbalances in females (Sulman 1980; van Casteren et al. 2021).

The pathogenesis of migraine is complex, and the possible factors causing provoking migraine attacks include atmospheric conditions, diet, alcohol, endogenous hormonal influences, and menstruation, along with synthetic alkaloids and irritants, such as volatile molecules, stress, malaise, drugs, smoking, odor, light, sound, and sleep disturbances (Kesserwani 2021; Marmura 2018; Mayans and Walling 2018). Previous studies have reported that the factors associated with migraines include weather changes, barometric pressure, maximum instantaneous wind speed, temperature difference, mean wind speed, humidity, warm climatic conditions (higher than usual), temperature changes, and photophobia (Katsuki et al. 2023; Scheidt et al. 2013). In the study, an association between weather changes and migraine attacks was suggested, where the meteorological data were collected in Utsunomiya, Japan, and compared with headache diaries of 28 Japanese patients (Kimoto et al. 2011). In contrast, some studies have raised doubts about the influence of weather factors on the onset of migraines and have shown that the effect of the former is modest, and low pressure is not directly linked to migraine attacks (Tanik, Saçmaci, and Aktürk 2020; Zebenholzer et al. 2011). Furthermore, the results on review of recent studies were found to be inconsistent and estimated that weathers’ effect on migraine attacks is around 20% and concluded that very strong weather factors have a more significant effect on migraine attacks (Denney, Lee, and Joshi 2024). Hence, the findings of the studies in relation to weather changes and migraine attacks have been found to be controversial, and it is necessary to perform further studies to conclude an association between weather factors and the onset of migraine. Further, seasonality influences human bodies, and people tend to adapt to weather changes by altering their way of living. According to the Japan Meteorological Agency, compared with other countries in the world, Japan has four distinct seasons (spring, summer, autumn, and winter), articulated within a complex climate system, characterized by meteorological differences in temperatures, atmospheric pressure, humidity, and wind speeds in different regions of Japan, mainly because of the country's length, geographical disposition of the islands, and highly mountainous topography (Japan Meteorological Agency 2024). Hence, we proposed to examine migraine onset with seasons in Japan. Most of the previous studies focused on weather factors rather than on the seasons; hence, there is a need for further studies to investigate the relationship between potential seasonal variations and the onset of migraine attacks, which will eventually help to determine high‐risk seasons for migraines and mitigate onsets.

Therefore, this study aims to identify seasonal differences in migraine attacks specific to certain regions in Japan and hypothesize that variations in seasonality in Japan are associated with the onset of migraine.

## 2 Research Methods

### 2.1 Data Sources

A nationwide time‐series analysis was conducted by analyzing a dataset comprising health insurance claims data from JMDC Inc. database (JMDC data) (Kumamaru et al. 2023; Nagai et al. 2020) and city‐level meteorological data collected by the Japan Weather Association between January 2018 and December 2021. The JMDC data allowed us to track all the medical treatment that the patients had received, including different facilities while on the same health insurance. All the patients included in the database were under 75 years old and covered by the insurance. The Japan Weather Association has a service to provide weather data called “HealthWeather,” which can be combined with JMDC data for research purposes. Until now, the former has been used in some epidemiological studies (Sanada et al. 2020). This study has been approved by the ethics review committee of the research division of Otsuka Pharmaceuticals Co. Ltd (approval number #220621). For the use of these combined datasets, informed consent was waived because they were anonymized.

### 2.2 Participants

Among the participants who were continuously registered in the database between 2018 and 2021 (data period), we first selected patients diagnosed with migraine at least once in each data period. After, we enrolled patients who had been confirmed to be registered in the prior January of each year. Excluding the influence of coronavirus infection, we eventually used patients from January 2018 to December 2019 (study period). We included the patients with at least one confirmed diagnosis of migraine throughout the whole data period (Yamato et al. 2023) and the following in the assessment window (January to December in each year): migraine (G43); cluster headaches and other trigeminal autonomic cephalgias (TAC) (G44.0); vascular headache, not classified elsewhere (G44.1); tension‐type headache (G44.2); posttraumatic headache (G44.3); drug‐induced headache, not elsewhere classified (G44.4); and other specified headache syndromes (G44.8). Then we ruled out patients aged < 18 years, enrolled at the end of December of each year, those who received triptans or ergotamines for a total of 120 tablets in the assessment window, those with comorbidities that caused headache (i.e., secondary headache), and those who were not prescribed triptans in the prior year of each year. The remaining patients were included in the subsequent analyses, and they were divided into four groups according to seasons: winter (January–March), spring (April–June), summer (July–September), and autumn (October–December) (Figure S1). We did not calculate an accurate sample size and included as many samples as possible from the database.

### 2.3 Outcome Measures

The seasonal prescribing patterns of triptans were used as a measure to assess seasonal migraine attacks. Triptan prescriptions were determined as the number of triptans (tablets/month), which was calculated as a total dose of triptans divided by the number of months in each season in 2019. The triptan dosage was defined as a prescription amount of triptans in each season, and a measured dose was converted into a sumatriptan succinate equivalent based on a single dose per administration and a maximum daily dose of each medication (Table S5).

### 2.4 Study Variables

The following covariates were determined in the study period for up to a year prior to the first month of each season: sex, age, drugs used to treat acute migraine symptoms, drugs used to prevent migraine, concomitant medications, comorbidities that may trigger or exaggerate migraine, and others (Figure S1). Female patients were categorized into either ≤ 51 or ≥ 52 years old age groups according to the estimated menopause age (Uemura et al. 2012). The clinical practice guidelines for headache defined migraine medications and concomitant medications. Acute migraine medications included triptans, and we also included anxiolytics, NSAIDs, steroids, ergometrine, and others (tramadol) (Table S1), and preventive medications covered antiepileptics, antidepressants, β‐blockers, calcium channel blockers, and ARB/ACEi (Table S2). The acute migraine medications except triptan and preventive medications mentioned are permitted for off‐label use based on the Headache Treatment Guidelines 2021 (Shibata 2023). We included the comorbidities defined by previous literature (Gerstl et al. 2021; Kim et al. 2018): insomnia not caused by a substance or known physiological condition (F51), sleep disorders (G47), mood disorders (F30–F39), and anxiety disorders (F40, F41) as a proxy for stress, pain, and the other conditions associated with female genital organs and the menstrual cycle (N94), hypothyroidism (E03.9), and allergic contact dermatitis caused by other agents (L23.8).

The following weather covariates comprised the analysis conducted for all days in 2019 by region in Japan: temperature, atmospheric pressure, relative humidity, maximum instantaneous wind, and sunshine duration. Japan is a string of islands located amid the North Pacific Ocean and Sea of Japan toward the east of the Peninsula of Korea with a geographical alignment of approximately 20°–45° north latitude and between approximately 123° and 154° east longitude, the characteristics of Japan's climatic conditions are a bit diverse. According to the Koppen–Geiger climate classification of Japan, Northern Japan (Hokkaido and the north of Tohoku on Honshu Island) and Eastern Japan (such as south of Tohoku, Kanto, and Chubu regions on Honshu Island) have a temperate and humid continental climate (Dwb and Dfb, respectively), and Western regions (Kansai onward) have a warm and humid continental climate (Dfa), whereas Southern Japan (south Kyushu to Taiwan) has a warm subtropical oceanic climate (Cfa) (Beck et al. 2018). The eight variables that refer to regions (Hokkaido, Tohoku, Kanto, Chubu, Kinki, Chugoku, Shikoku, and Kyushu) in Japan are shown in Table S3.

### 2.5 Statistical Analysis

Study participants’ characteristics were summarized in the groups outlined by seasons, where triptan prescription amounts were compared across seasons and regions.

We evaluated seasonal changes in migraine onset using linear mixed effect models for repeated measures (LMMRMs), adjusted for sex (male/female), age (continuous variable), menopause category by age (≤ 52 or ≥ 52 years), acute medications for migraines, medications that prevent migraines, the comorbidities linked to increased migraine risks, and the other comorbidities (Model 1). Additionally, eight variables representing regions were employed to assess the impact of the latter on the previous model (Model 2). All analyses were conducted with SAS statistical software version 9.4 (SAS Institute Inc., Cary, NC, USA). This study was reported in accordance with the STROBE and RECORD‐PE Checklists.

## 3 Results

**FIGURE 1:**
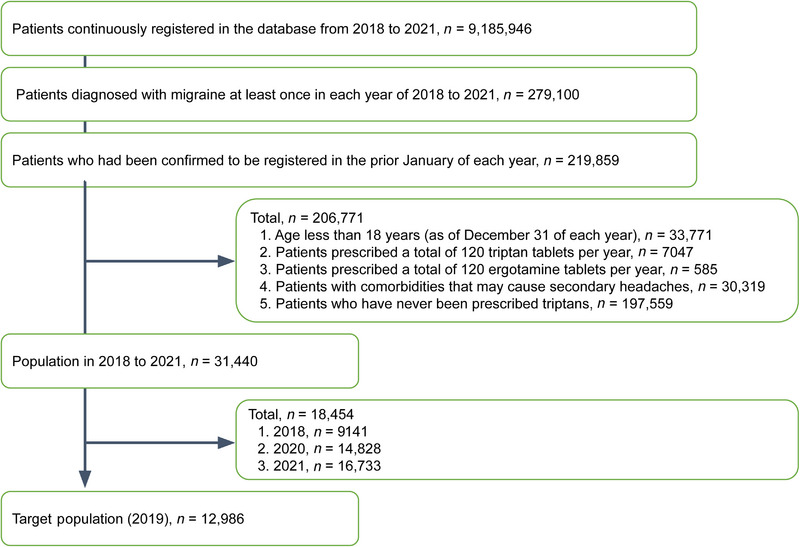
Flow chart of the target population in this study.

The participants’ selection for this study is reflected in Figure 1. Among the 9,185,946 patients recorded in the JMDC database in 2018–2021, 279,100 patients were diagnosed with migraine during the data period. Those who met at least one of the exclusion criteria were eliminated, and 31,440 patients were enrolled. Due to the coronavirus infection, we focused on patients in 2019, and 12,986 were finally analyzed.

### 3.1 Patient Characteristics

In this study, 74.1% of the sample comprised females, with a mean (standard deviation) age of 44.1 (10.6) years (Table 1). Patients aged ≤ 51 years accounted for 81.0% of the total number of patients. Acute medications commonly prescribed for migraine included triptan (96.1%–99.9%), acetaminophen/NSAIDs (75.9%–76.3%), anxiolytics/antipsychotics/anesthetics/antiemetics (30.6%–31.5%), followed by steroids (8.9%–9.0%), others (8.9%–9.5%), and ergotamine (0.8%–0.9%). The medications prescribed frequently to prevent migraine were calcium channel blockers (2.7%), antiepileptic drugs (13.9%–14.4%), and antidepressants (11.9%–12.1%). The most common comorbidities that could potentially induce the onset of migraines were sleep disorder (17.3%–18.2%), mood disorder (15.2%–15.7%), anxiety disorder (9.6%–10.1%), and dysmenorrhea (7.4%–7.7%); other frequent comorbidities were hypertension (11.1%–12.0%) and epilepsy (5.3%–5.4%).

**TABLE 1:** Characteristics of the patients with migraine diagnosed and treated in each season (2019).

	Whole season in 2019 (*n* = 12,986)			
	Winter (Jan–Mar)	Spring (Apr–June)	Summer (July–Sep)	Autumn (Oct–Dec)
Sex (Female)	9622 (74.1)			
Age (Mean, SD)	44.1 (10.6)			
Female age group (Age ≤ 51 years)	10,516 (81.0)			
Acute medication use to migraine				
Any	12,982 (100.0)	12,974 (99.9)	12,946 (99.7)	12,903 (99.4)
Triptan	12,970 (99.9)	12,915 (99.5)	12,758 (98.2)	12,474 (96.1)
Acetaminophen/NSAIDs	9853 (75.9)	9853 (75.9)	9855 (75.9)	9850 (75.9)
Anxiolytics/antipsychotics/etc.	4088 (31.5)	4073 (31.4)	4085 (31.5)	3968 (30.6)
Steroid	1164 (9.0)	1171 (9.0)	1158 (8.9)	1162 (8.9)
Others	1152 (8.9)	1165 (9.0)	1206 (9.3)	1236 (9.5)
Ergotamine	113 (0.9)	112 (0.9)	118 (0.9)	104 (0.8)
Medication use to prevent migraine				
Any	5248 (40.4)	5253 (40.5)	5271 (40.6)	5269 (40.6)
Antiepileptic drugs	1864 (14.4)	1866 (14.4)	1855 (14.3)	1802 (13.9)
Other concomitant medications	1837 (14.1)	1842 (14.2)	1866 (14.4)	1904 (14.7)
Antidepressants	1548 (11.9)	1575 (12.1)	1576 (12.1)	1548 (11.9)
Beta‐blockers	1441 (11.1)	1441 (11.1)	1445 (11.1)	1454 (11.2)
Calcium channel blockers	346 (2.7)	349 (2.7)	348 (2.7)	345 (2.7)
ARB/ACE inhibitors	285 (2.2)	294 (2.3)	288 (2.2)	293 (2.3)
The comorbidities linked to increased migraine risk				
Any	4297 (33.1)	4359 (33.6)	4441 (34.2)	4478 (34.5)
Sleep disorder	2241 (17.3)	2262 (17.4)	2333 (18.0)	2367 (18.2)
Mood disorder	1969 (15.2)	2001 (15.4)	2037 (15.7)	2043 (15.7)
Anxiety disorder	1243 (9.6)	1253 (9.6)	1285 (9.9)	1306 (10.1)
Dysmenorrhea	959 (7.4)	971 (7.5)	987 (7.6)	997 (7.7)
Light sensitivity	2 (0.0)	3 (0.0)	3 (0.0)	4 (0.0)
Hypothyroidism	0 (0.0)	0 (0)	0 (0.0)	0 (0.0)
Other comorbidities				
Any	2074 (16.0)	2119 (16.3)	2167 (16.7)	2191 (16.9)
Primary essential hypertension	1444 (11.1)	1487 (11.5)	1530 (11.8)	1552 (12.0)
Epilepsy	686 (5.3)	687 (5.3)	697 (5.4)	7.1 (5.4)
Restless legs syndrome	64 (0.5)	63 (0.5)	67 (0.5)	69 (0.5)
Asthma	40 (0.3)	39 (0.3)	38 (0.3)	35 (0.3)

*Note*: The values are numbers (percentages) unless otherwise stated.

### 3.2 Weather Conditions by Region

Weather conditions in each region are demonstrated in Table S4. Eligible patients comprised higher percentages in Kanto (42.5%), Chubu (26.6%), and Kinki (12.3%) than in any other region. The average temperature was 26.0°C in summer and 7.1°C in winter, and the average atmospheric pressure was the highest in autumn (1019.1 hPa) and the lowest in summer (1010.3 hPa) in all regions.

### 3.3 Triptans Prescription Amount by Season and Region

The mean prescription amount of triptans was 2.12 tablets/month in spring and summer, 2.09 tablets/month in autumn, and 2.00 tablets/month in winter. Some seasonal and regional differences in this parameter. The highest amount was reported to be administered in autumn in Hokkaido (2.45 tablets/month), and the lowest amount was reported in spring in Shikoku (1.59 tablets/month) (Table 2). The geographical distribution of triptan prescription in each season is shown in Figure S2.

**TABLE 2:** The prescription amounts of triptans in each season by region in Japan.

	Region in Japan								
	Whole Japan	Hokkaido	Tohoku	Kanto	Chubu	Kinki	Chugoku	Shikoku	Kyusyu
Season/No. of patients	(*n* = 12,986)	(*n* = 228)	(*n* = 678)	(*n* = 5520)	(*n* = 3458)	(*n* = 1594)	(*n* = 618)	(*n* = 171)	(*n* = 719)
Winter (Jan–Mar)	2.00 (2.40)	2.27 (2.84)	1.86 (2.44)	1.98 (2.19)	2.08 (2.71)	1.95 (2.48)	2.10 (2.33)	1.90 (2.30)	1.86 (1.99)
Spring (Apr–Jun)	2.12 (2.44)	2.37 (2.81)	1.82 (2.19)	2.08 (2.27)	2.24 (2.74)	2.18 (2.54)	2.20 (2.48)	1.59 (1.98)	1.91 (2.07)
Summer (Jul–Sep)	2.12 (2.53)	2.32 (3.25)	1.92 (2.42)	2.10 (2.38)	2.21 (2.80)	2.04 (2.49)	2.30 (2.46)	1.91 (2.48)	1.98 (2.24)
Autumn (Oct–Dec)	2.09 (2.59)	2.45 (3.06)	1.88 (2.46)	2.07 (2.40)	2.21 (2.92)	2.06 (2.60)	2.12 (2.47)	1.88 (2.36)	1.92 (2.26)

*Note*: The numbers (mean, [SD]) were derived from the following formula: total volume of triptan prescription/3 months (the number of months in the season).

### 3.4 Seasonal Variations in Triptan Prescriptions

In Model 1, where LMMRM was incorporated, the number of triptans in spring was compared with those in summer (*β* = 0.003; 95% confidence interval [CI]: −0.047 to 0.053), autumn (*β *= −0.016; 95% CI: −0.063 to 0.031), and winter (*β *= −0.117; 95% CI: −0.169 to −0.065) (Table 3). The analysis in Model 2 included eight regional variables, resulting in values similar to those obtained in Model 1 for the parameters: summer (*β *= 0.004; 95% CI: −0.046 to 0.054), autumn (*β* = −0.016; 95% CI: −0.063 to 0.033), and winter (*β* = −0.117; 95% CI: −0.168 to −0.066).

**TABLE 3:** The association between seasons and prescription amounts of triptans.

	Model 1			Model 2 (considered with regional variables)		
Variables	*β* ^a^	95% CI	*p* value	*β* ^b^	95% CI	*p* value
Winter (Jan–Mar)	−0.117	−0.169 to −0.065	< 0.001	−0.117	−0.168 to −0.066	< 0.001
Spring (Apr–June)[Reference]						
Summer (July–Sep)	0.003	−0.047 to 0.053	0.898	0.004	−0.046 to 0.054	0.885
Autumn (Oct–Dec)	−0.016	−0.063 to 0.031	0.504	−0.016	−0.063 to 0.033	0.543

Abbreviation: CI, confidence interval.

^a^ Beta coefficient is triptan prescription volume calculated from repeated measures linear mixed effect models, adjusting sex (male/female), age(continuous), age category (< 52 or ≥ 52 years), acute medications for migraine, medications to prevent migraine, comorbidities linked to increased migraine risk, and other comorbidities.

^b^ Eight regional variables (Hokkaido, Tohoku, Kanto, Chubu, Kinki, Chugoku, Shikoku, and Kyusyu) were added to Model 1.

## 4 Discussion

We investigated seasonal variations in migraine onset by examining the prescription amount of triptans prescribed between seasons and regions in Japan. The analyses in Model 1 and Model 2 revealed that from autumn to winter, the prescription amount was lower than in spring. The results obtained imply that there were significant differences in migraine onsets between seasons, along with confounding factors such as age, sex, concomitant medications, comorbidities, and regions. Our results are supported by those of previous studies (Katsuki et al. 2023, Li et al. 2019; Scheidt et al. 2013), which cast doubt on a significant correlation between air temperature and migraines as well as a negative correlation between humidity and migraines. Our weather data demonstrated that winter produces the lowest average temperature and relative humidity of the year (Table S4).

According to our results, the negative association between seasons and migraines was not statistically significant in autumn, whereas it was characterized by the highest average atmospheric pressure. This finding may be in line with those of the previous studies that did not show a relationship between atmospheric pressure and migraines (Akgün et al. 2021; Kimoto et al. 2011).

This study showed that the highest prescription amount of triptans was found to be in Hokkaido (2.45 tablets/month) in autumn and that the lowest was in Shikoku in spring (1.59 tablets/month). The regional difference in the prescription of triptan may be a result of factors such as the concentration of headache specialists, meteorological influences, and age demographics. However, in the LMMRM analysis, there were no changes in Model 2 adjusted by region from Model 1. The results suggest that clinicians take seasonality, not localization, into account for implementation of migraine treatment so that the results can be valuable for preventive care for patients with migraines.

There are several limitations in this study. First, although our current results were received through analyses involving the data collected nationwide, weather variables may not precisely reflect the weather conditions to which the patients were exposed, as we used the weather characteristics of the places where the patients had treatment for migraines. Second, one of the strengths of this study is the use of an insurer database, which allows us to capture all prescription information from patients, but it does not provide information on over‐the‐counter (OTC) drugs. However, in Japan, there are no OTC acute medications for migraine, including triptans, and therefore we do not expect the used data on these types of medications to have any influence on the result outcomes of this study. Third, as with any observational study, we could not eliminate the possibility of unmeasured confounders that may be associated with migraines, such as odor, noise, and diet. Finally, the data on medical claims used in this study were obtained from insurers for workers and their families and did not include patients enrolled in region‐based insurance for those who were unemployed or self‐employed. Additionally, the database does not cover the population group of age over 75 years old. However, the morbidity of migraines is mainly reported among the population in the 30s; therefore, it may not have a critical impact on this study.

## 5 Conclusion

Using data on large‐scale nationwide medical claims combined with that of weather in Japan, the present study demonstrated seasonal differences in triptan prescription patterns, suggesting seasonal changes in migraine attacks. Our findings suggest that clinicians should recommend that patients start preventive actions, including self‐care and drug therapies, in the low‐risk season (winter) in order to reduce migraine onsets in the high‐risk season (spring) regardless of region.

## Author Contributions

**Muneto Tatsumoto**: conceptualization, methodology, supervision, writing–review and editing, validation. **Koichi Hirata**: conceptualization, methodology, supervision, writing–review and editing, validation. **Takeo Nakayama**: conceptualization, methodology, supervision, writing–review and editing, validation. **Kentaro Yamato**: conceptualization, methodology, writing–review and editing, supervision, writing–original draft, project administration, funding acquisition, investigation, data curation, visualization, formal analysis, resources, validation, software. **Hiromi Sano**: conceptualization, methodology, writing–review and editing, writing–original draft, supervision, project administration, funding acquisition, investigation, data curation, visualization, formal analysis, resources, validation, software. **Lyo Inuyama**: conceptualization, supervision, resources, funding acquisition.

## Conflicts of Interest

K.Y., H.S., and L.I. are Otsuka Pharmaceutical Co. Ltd (Japan) employees. K.H., M.T., and T.N. received consulting fees from Otsuka Pharmaceutical Co. Ltd during the conduct of the study.

### Peer Review

The peer review history for this article is available at https://publons.com/publon/10.1002/brb3.70184.

## Supporting information




**Supplementary Table 1**. Definition of acute treatment for migraine.
**Supplementary Table 2**. Definition of medicines used for prophylactic treatment for migraine.
**Supplementary Table 3**. Definition of the eight regions in Japan.
**Supplementary Table 5**. Calculation of sumatriptan succinate equivalent.

**Supplementary Figure 1**. Study design diagram.

**Supplementary Figure 2**. Geographical distribution of triptan prescription in each season.

**Supplementary Table 4**. Weather conditions in each season by region in Japan.

## Data Availability

The data that support the findings of this study are available from JMDC Inc. but were used under license for the current study; therefore, restrictions apply, and the data are not publicly available. For inquiries about access to the data set used in this study, please contact JMDC (https://www.jmdc.co.jp).
