# Supplementary material for: Seasonal Variations in Triptan Prescription in Japan: A Nationwide Time‐Series Analysis
Source: Brain Behav. 2024 Dec 22;14(12):e70184. doi: 10.1002/brb3.70184 (PMC11663837; doi:10.1002/brb3.70184)
Supplement: Supplementary file 1 — Supplementary Table 1. Definition of acute treatment for migraine. Supplementary Table 2. Definition of medicines used for prophylactic treatment for migraine. Supplementary Table 3. Definition of the eight regions in Japan. Supplementary Table 5. Calculation of sumatriptan succinate equivalent. [file BRB3-14-e70184-s002.docx]

# **Supporting Information**

Title:

Seasonal variations in triptan prescription in Japan: a nationwide time-series analysis

# **List of supporting information**

[Supporting Information 1](#_heading=h.gjdgxs)

[List of supporting information 1](#_heading=h.30j0zll)

[Supplementary Table 1. Definition of acute treatment for migraine 1](#_heading=h.1fob9te)

[Supplementary Table 2. Definition of medicines used for prophylactic treatment for migraine 4](#_heading=h.3znysh7)

[Supplementary Table 3. Definition of the eight regions in Japan 6](#_heading=)

[Supplementary Table 4. Definition of the eight regions in Japan 6](#_heading=h.gl8tmvfqy7ia)

# **Supplementary Table 1. Definition of acute treatment for migraine**

| Drugs | ATC codes |
| --- | --- |
| Triptan | N02CC01 Sumatriptan |
|  | N02CC02 Naratriptan |
|  | N02CC03 Zolmitriptan |
|  | N02CC04 Nizatriptan |
|  | N02CC06 Eletriptan |
| Anxiolytics | A03FA01 Metoclopramide |
|  | A03FA03 Domperidone |
|  | N05AB04 Prochlorperazine |
|  | N05AA01 Chlorpromazine |
|  | N05AD08 Droperidol |
|  | N01AX10 Propofol |
|  | N05BA01 Diazepam |
| Acetaminophen, NSAIDs | N02BE01 Acetaminophen |
|  | N02BA01 Acetylsalicylic acid |
|  | M01AE01, M02AA13 ib uprofen |
|  | M01AE02, M02AB4412 Naproxen |
|  | M01AB05, M02AA15 Diclofenac |
|  | M01AB01 Indomethacin |
|  | M01AB11 Acemetacin |
|  | M01AH01 Celecoxib |
|  | M01AB08 Etodolac |
|  | M01AG01 Mefe namic acid |
|  | M02AA31 Loxoprocin |
|  | M01AC05 Lornoxicam |
| Ergotamine | N02CA01 Dihydroergotamine compound |
|  | N02CA02 Ergotamine |
|  | N02CA51 Dihydroergotamine compound |
|  | N02CA52 Ergotamine other than psychosuppressive drugs |
|  | N02CA52 Ergotamine psychosuppressive combination |
| Other | N02AX02, N02AJ13 to 16 Tramadol (fixed dose) |
|  |  |
|  |  |
|  |  |
|  |  |

# **Supplementary Table 2. Definition of medicines used for prophylactic treatment for migraine**

| Drugs | ATC codes |
| --- | --- |
| Antiepileptic agent | N03AG01 Valproic acid |
|  | N03AX11 Topiramate |
|  | N03AX12 Gabapentin |
|  | N03AX14 Levetiracetam |
| Antidepressant | N06AA09; N06CA01 Amitriptyline |
|  | N06AA01 Nortriptyline |
|  | N06AA02 Imipramine |
|  | N06AA04 Clomipramine |
|  | N06AX05 Trazodone |
|  | N06AX03 Mianserin |
|  | N06AB08 Iboxamine |
|  | N06AB05 Paroxetine |
|  | N05AL01 Sulpiride |
|  | N06AX21 Duloxetine |
| Calcium channel blocker | C08DA01 Verapamil |
|  | C08DB01 Diltiazem |
|  | C08CA04 Nicardine |
| ARB/ACE inhibitor | C09CA06 Candesartan |
|  | C09AA03 Lisinopril |
|  | C09AA02 Enalapril |
|  | C09CA08 Olmesartan |
| Other | D00783 Botulinum toxin type A |
|  | N02CX05 Dimethothiazine |
|  | A06AD01 to 04 and A06AD19 antacid |
|  | B2A11HA04 Riboflovin |
|  | M03BX02 Tizanidine |
|  | N05CH01 Melatonin |
|  | N05AH03 Olanzapine |
|  |  |
|  |  |
|  |  |

#

# **Supplementary Table 3. Definition of the eight regions in Japan**

| Region | Name of Prefecture |
| --- | --- |
| Hokkaido | Hokkaido |
| Tohoku | Aomori, Iwate, Akita, Miyagi, Yamagata, Fukushima |
| Kanto | Ibaraki, Tochigi, Gunma, Saitama, Chiba, Tokyo, Kanagawa |
| Chubu | Niigata, Toyama, Ishikawa, Fukui, Yamanashi, Nagano, Gifu, Shizuoka, Aichi |
| Kinki | Mie, Shiga, Kyoto, Osaka, Hyogo, Nara, Wakayama |
| Chugoku | Tottori, Shimane, Okayama, Hiroshima, Yamaguchi |
| Shikoku | Tokushima, Kagawa, Ehime, Kochi |
| Kyushu | Fukuoka, Saga, Nagasaki, Kumamoto, Oita, Miyazaki, Kagoshima, Okinawa |

**Supplementary Table 4. Weather conditions in each season by region in Japan**

# **Supplementary Table 5. Calculation of sumatriptan succinate equivalent**

| Brand Name (General Name) | Formulation | Single Dose | Total Daily Dose | Conversion Factor 1 | Conversion Factor 2 |
| --- | --- | --- | --- | --- | --- |
| Imigran^®^ (Sumatriptan) | Tablet, Oral Liquid | 50mg | 200mg | 1 | 1 |
|  | Injection, Subcutaneous injection | 3mg | 6mg | 16.66667 | 33.33333 |
|  | Nasal Spray | 20mg | 40mg | 2.5 | 5 |
| Zomig^®^ (Zolmitriptan) | Tablet, RM Tablet, OD Tablet | 2.5mg | 10mg | 20 | 20 |
| Relpax^®^ (Eletriptan) | Tablet | 20mg | 40mg | 2.5 | 5 |
| Maxalt^®^ (Rizatriptan) | Tablet, RM Tablet, OD Tablet | 10mg | 20mg | 5 | 10 |
| Amerge^®^ (Naratriptan) | Tablet | 2.5mg | 5mg | 20 | 40 |

**Supplementary Figure 1. Study design diagram**

**Supplementary Figure 2. Geographical distribution of triptan prescription in each season**
