# Supplementary figures and images for: Seasonal Variations in Triptan Prescription in Japan: A Nationwide Time‐Series Analysis
Source: Brain Behav. 2024 Dec 22;14(12):e70184. doi: 10.1002/brb3.70184 (PMC11663837; doi:10.1002/brb3.70184)

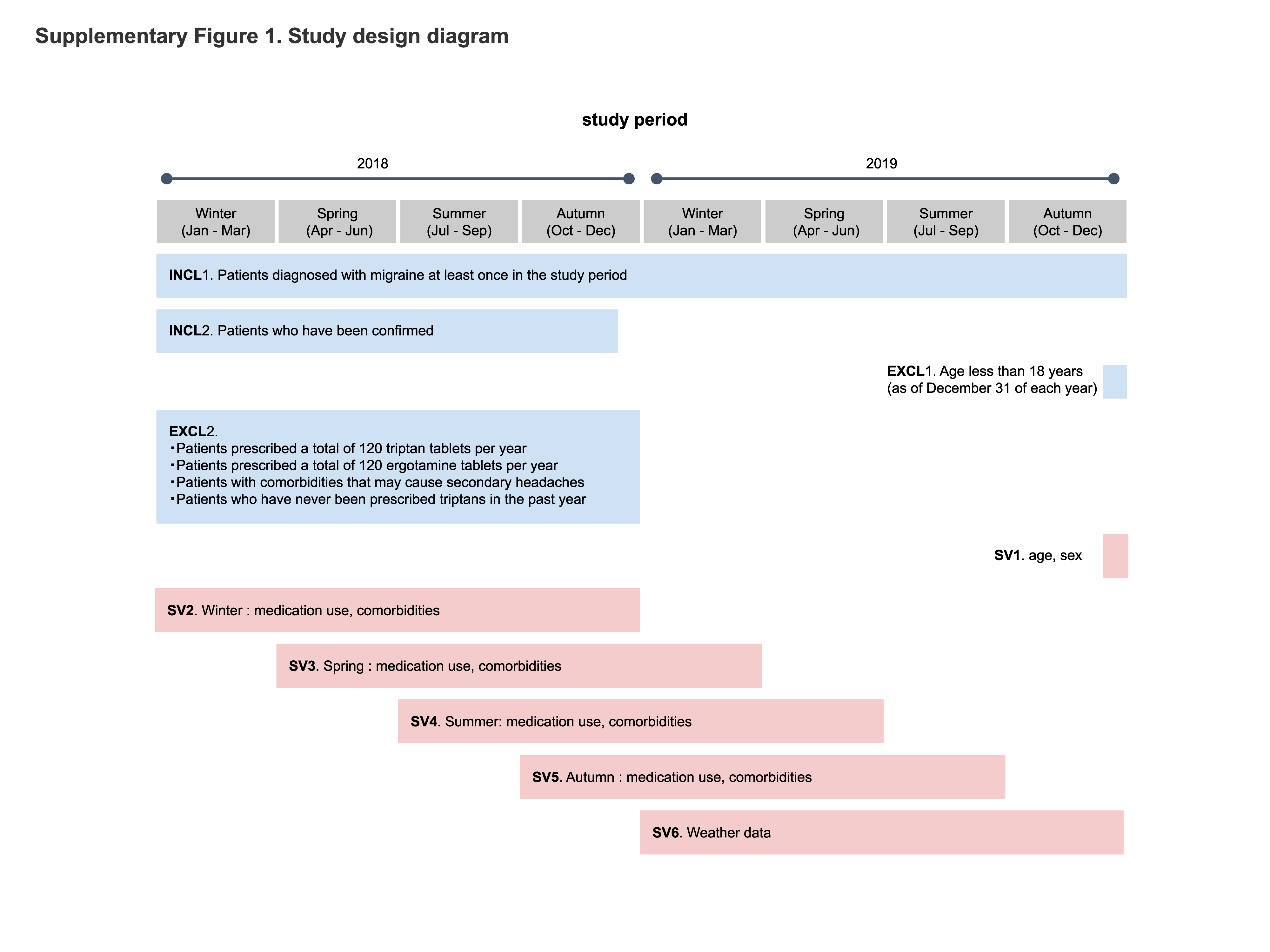

Supplement: Supplementary file 2 — Supplementary Figure 1. Study design diagram. [file BRB3-14-e70184-s004.tif]

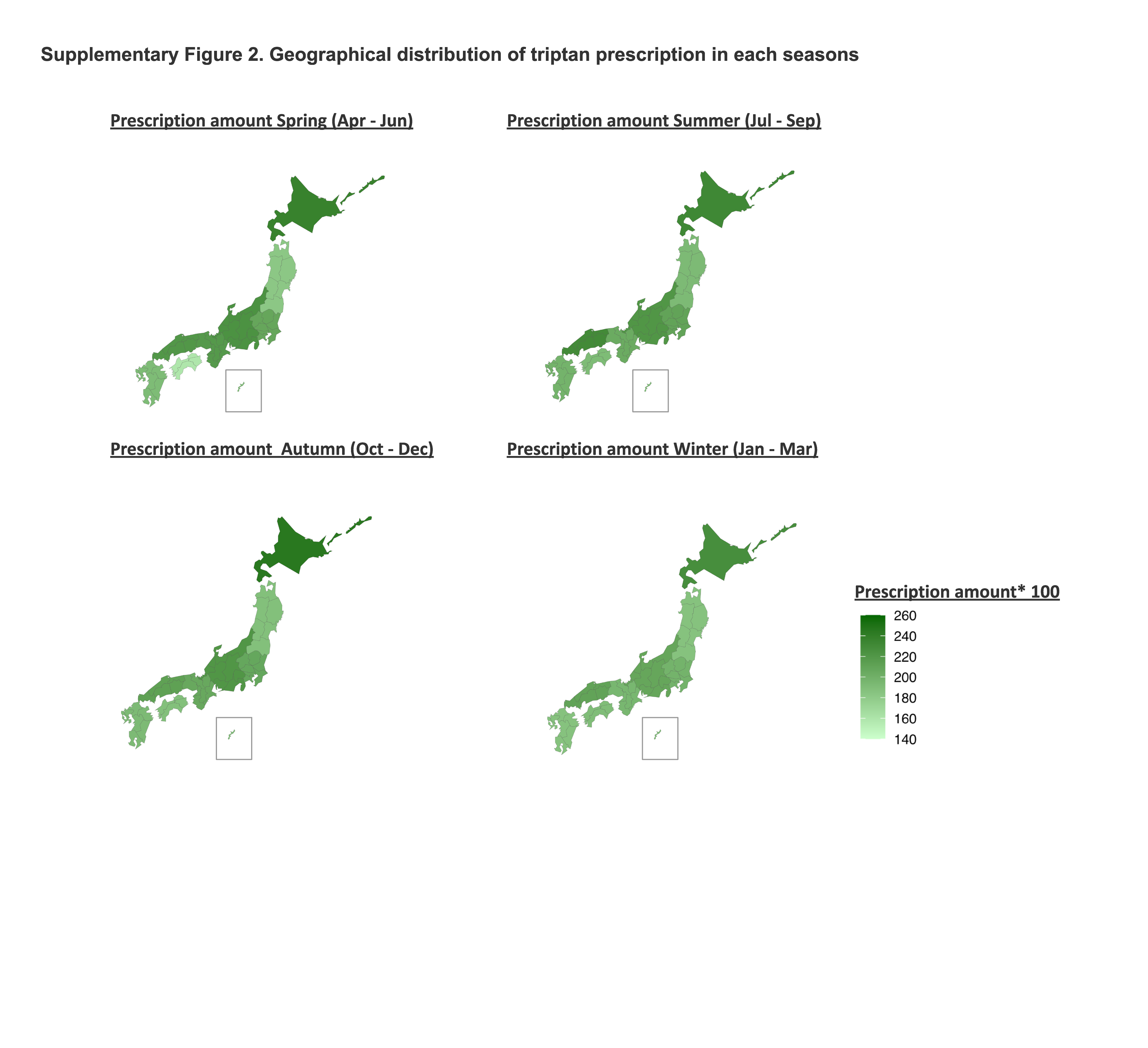

Supplement: Supplementary file 3 — Supplementary Figure 2. Geographical distribution of triptan prescription in each season. [file BRB3-14-e70184-s003.tif]
